# Writing the Future: Artificial Intelligence, Handwriting, and Early Biomarkers for Parkinson’s Disease Diagnosis and Monitoring

**DOI:** 10.3390/biomedicines13071764

**Published:** 2025-07-18

**Authors:** Giuseppe Marano, Sara Rossi, Ester Maria Marzo, Alice Ronsisvalle, Laura Artuso, Gianandrea Traversi, Antonio Pallotti, Francesco Bove, Carla Piano, Anna Rita Bentivoglio, Gabriele Sani, Marianna Mazza

**Affiliations:** 1 Unit of Psychiatry, Fondazione Policlinico Universitario Agostino Gemelli IRCCS, 00168 Rome, Italymariannamazza@hotmail.com (M.M.); 2 Department of Neurosciences, Università Cattolica del Sacro Cuore, 00168 Rome, Italy; francesco.bove@policlinicogemelli.it (F.B.); annarita.bentivoglio@policlinicogemelli.it (A.R.B.); 3 Accademia di Psicologia e Espressione Della Scrittura, 00168 Rome, Italy; 4 Unit of Medical Genetics, Department of Laboratory Medicine, Ospedale Isola Tiberina-Gemelli Isola, 00186 Rome, Italy; gianandrea.traversi@gmail.com; 5 Department of Management and Law, University of Rome “Tor Vergata”, 00133 Rome, Italy; 6 Neurology Unit, Fondazione Policlinico Universitario Agostino Gemelli IRCCS, 00168 Rome, Italy

**Keywords:** Parkinson’s Disease, handwriting, artificial intelligence, machine learning, early diagnosis, biomarkers, Motor Disorders, digital health, telemedicine, personalized medicine

## Abstract

Parkinson’s disease (PD) is a progressive neurodegenerative disorder that impairs motor function, including the fine motor control required for handwriting. Traditional diagnostic methods often lack sensitivity and objectivity in the early stages, limiting opportunities for timely intervention. There is a growing need for non-invasive, accessible tools capable of capturing subtle motor changes that precede overt clinical symptoms. Among early PD manifestations, handwriting impairments such as micrographia have shown potential as digital biomarkers. However, conventional handwriting analysis remains subjective and limited in scope. Recent advances in artificial intelligence (AI) and machine learning (ML) enable automated analysis of handwriting dynamics, such as pressure, velocity, and fluency, collected via digital tablets and smartpens. These tools support the detection of early-stage PD, monitoring of disease progression, and assessment of therapeutic response. This paper highlights how AI-enhanced handwriting analysis provides a scalable, non-invasive method to support diagnosis, enable remote symptom tracking, and personalize treatment strategies in PD. This approach integrates clinical neurology with computer science and rehabilitation, offering practical applications in telemedicine, digital health, and personalized medicine. By capturing dynamic features often missed by traditional assessments, AI-based handwriting analysis contributes to a paradigm shift in the early detection and long-term management of PD, with broad relevance across neurology, digital diagnostics, and public health innovation.

## 1. Introduction

Parkinson’s disease (PD) is a progressive neurodegenerative disorder that primarily affects the motor system, but its consequences extend far beyond movement dysfunction. Caused by the loss of dopaminergic neurons in the substantia nigra, PD manifests through hallmark motor symptoms such as bradykinesia, rigidity, resting tremor, and postural instability. However, an increasing body of research recognizes the multifaceted nature of PD, with non-motor symptoms, including cognitive decline, mood disorders, sleep disturbances, and autonomic dysfunction, emerging as critical components of the disease burden [1,2]. These symptoms can precede the motor signs by years, often complicating early diagnosis and delaying treatment.

Among the earliest and most characteristic motor manifestations of PD is the deterioration of handwriting. This fine motor skill is frequently impaired in PD due to bradykinesia, rigidity, and tremor, resulting in progressively smaller, slower, and less legible writing—a condition known as micrographia [3]. While often considered a visible marker of motor decline, micrographia may also signal early-stage PD and reflects deeper deficits in motor planning and neuromotor coordination related to dopaminergic dysfunction [3,4]. Beyond its clinical significance, the loss of handwriting ability is emotionally impactful. It affects personal expression and communication, potentially leading to frustration, loss of autonomy, and psychological distress [5,6,7].

Efforts to rehabilitate handwriting impairments in PD have included behavioral therapies, such as the Lee Silverman Voice Treatment (LSVT) BIG program, which aims to improve movement amplitude, with some success reported in handwriting performance [8]. Assistive technologies, ranging from ergonomic writing tools to speech-to-text apps, also offer practical support [9]. Yet, traditional clinical assessments of handwriting remain limited in their diagnostic utility. These methods rely on visual inspection or standardized tasks, often overlooking the dynamic elements of writing that are critical to motor function analysis.

PD diagnosis currently depends on clinical expertise and observation of motor symptoms, which introduces subjectivity and risk of misdiagnosis, particularly in early stages when symptoms may be subtle or non-specific [10,11]. Given the limitations of conventional approaches, there is growing interest in identifying objective biomarkers that can enhance diagnostic accuracy and enable earlier intervention. Among emerging technologies, artificial intelligence (AI), and more specifically machine learning (ML), has shown great promise in revolutionizing disease detection and monitoring [12,13,14,15].

ML algorithms applied to PD leverage diverse data sources, including voice analysis, gait patterns, neuroimaging, and, increasingly, handwriting data. These models offer the capacity to detect subtle abnormalities and discern complex patterns that may elude human evaluation [9,16]. However, the effectiveness of such models depends heavily on the quality and relevance of the extracted features. In this regard, static features such as handwriting size are often insufficient. Recent research has shifted focus toward dynamic, kinematic variables, including writing speed, pressure, fluency, and pen pauses, which better capture the underlying motor dysfunction and improve classifier performance [17].

The progression of PD necessitates continuous monitoring to tailor treatment and adjust therapeutic regimens over time. Non-motor symptoms, although often neglected, significantly influence quality of life and may respond differently to interventions. Standardized scales such as the Unified Parkinson’s Disease Rating Scale (UPDRS) and Parkinson’s Disease Questionnaire (PDQ-39) are widely used to track disease evolution, but their administration is resource-intensive and does not support real-time feedback [17,18].

Recent advances in wearable devices and digital health platforms have expanded the possibilities for continuous, remote assessment of motor symptoms [19,20]. These technologies enable passive data collection on tremor, gait, and other motor features outside the clinical setting, promoting personalized medicine and telemonitoring. In parallel, new diagnostic assays such as the alpha-synuclein seed amplification test offer molecular biomarkers for PD, although their widespread clinical use remains limited by invasiveness and cost [21].

Within this evolving landscape, AI-based handwriting analysis has emerged as a non-invasive, low-cost, and scalable strategy with unique advantages. Unlike traditional handwriting evaluation, AI-powered systems can process digital writing tasks captured through tablets or smartpens to extract rich temporal and spatial information. These tools can track disease onset and progression, assess treatment response, and even predict conversion from prodromal to clinical PD by analyzing subtle changes in handwriting patterns.

Despite this promise, several gaps remain in the literature. The integration of handwriting analysis with other digital biomarkers (e.g., gait and speech) is still in its infancy, limiting the development of holistic, multimodal diagnostic models. Standardized protocols for data acquisition, feature selection, and model validation are lacking, hampering reproducibility and clinical translation. In addition, many AI models suffer from limitations related to data diversity, algorithmic bias, and interpretability, factors critical for ethical deployment in healthcare.

In light of these challenges, the present review aims to provide a comprehensive and up-to-date synthesis of the current evidence on handwriting analysis in PD, with a particular focus on the role of artificial intelligence. We begin by examining the physiological basis and phenomenology of handwriting impairments in PD, including micrographia and dysgraphia. We then explore how dynamic features of handwriting serve as early digital biomarkers, followed by an overview of traditional and AI-based analytic techniques. Finally, we discuss the clinical applications of handwriting analysis, including early diagnosis, monitoring of disease progression, and evaluation of treatment response, while addressing the advantages and limitations of AI integration. By framing handwriting within a broader context of AI-enabled diagnostics, this paper contributes to ongoing efforts to enhance precision medicine and improve outcomes for individuals living with PD.

## 2. Handwriting Impairments in Parkinson’s Disease

### 2.1. Micrographia: Definition and Characteristics

An acquired reduction in handwriting size that results in smaller than typical letter strokes is known as micrographia [22]. The name was given by Pick in 1903 to a patient with a syphilitic infarct in the left thalamus [23], before Froment first associated it with Parkinson’s disease [24].

It is usually divided into two types: “consistent micrographia (CM)” and “progressive micrographia (PM)”. In contrast to PM, which is a serial decrease in handwriting size, CM refers to a global decrease in handwriting size compared to the handwriting before the onset of the disease [25]. Micrographia is a key feature of handwriting problems in Parkinson’s disease (PD) [26]. While both constant and progressive types are observed in PD, the latter is often considered a classical sign of the disease [27]. According to studies, micrographia may appear three to four years before other motor symptoms of Parkinson’s disease [28].

Understanding this enigmatic phenomenon is complicated by the fact that it can occur as part of the clinical picture of other neurodegenerative diseases, or in association with a structural lesion of the basal ganglia without PD [26].

The specific neurophysiological mechanisms that are impaired by dysfunction of the basal ganglia (BG) motor circuit and/or connectivity deficits between brain regions remain unclear. Furthermore, it is not well understood how these impairments contribute to the kinematic abnormalities observed in micrographia.

A recent fMRI study provided novel and comprehensive insights into the anatomical networks involved in micrographia [29]. In this study, right-handed Parkinson’s disease (PD) patients with either progressive or consistent micrographia, along with healthy controls, performed overlearned writing tasks while undergoing fMRI scans. The results showed that both PD patients and healthy controls activated similar brain regions during handwriting [29,30]. By analyzing brain region activations and assessing connectivity between different areas, the authors concluded that consistent and progressive micrographia are associated with different neural mechanisms.

In consistent micrographia, letter size correlated with activation of the left thalamus, left posterior putamen, and left caudal supplementary motor area (SMA), as well as connectivity between these regions. In progressive micrographia, Wu et al. observed a positive correlation between the degree of letter size decrement and activation in the left posterior putamen and left pre-SMA, similar to that observed in consistent micrographia. However, in progressive micrographia, letter size decrement was also positively correlated with activation in the right rostral cingulate motor area (rCMA) and negatively correlated with activation in the right cerebellum [29].

Levodopa was found to have a restorative effect on activation levels and connectivity patterns in consistent micrographia, probably due to the restoration of dopaminergic function in the BG motor circuit [29]. In contrast, the lack of improvement in progressive micrographia has been attributed to the inability of levodopa to repair the additional connectivity deficits. This variability in the efficacy of levodopa is consistent with clinical observations that only some PD patients recover letter size following dopaminergic therapy [31].

### 2.2. Kinematic and Dynamic Features Affected by PD

Advancements in handwriting research, particularly through the integration of graphic tablets, have enabled access to a range of dynamic, previously hidden variables related to handwriting movements. These variables, which go beyond the static visual trace, offer insight into the motor processes that underlie handwriting. Over the past decade, four primary variables have emerged as particularly informative in evaluating handwriting impairments in PD: size, duration, speed, and fluency [23]. A major focus of recent investigations has been to identify handwriting features that can effectively differentiate PD patients from healthy controls. However, many studies have yielded inconsistent findings. For instance, 43% of studies did not report significant differences in writing size between PD patients and age-matched controls, and 56% found no differences in writing duration [24]. In contrast, kinematic variables such as writing velocity and fluency have shown more reliable discriminative power. These features not only distinguish PD patients from controls but also reflect treatment states, such as differences between medicated and unmedicated conditions. These results suggest that handwriting impairment in PD cannot be solely attributed to micrographia, or reduced writing size, but instead reflects a broader spectrum of motor dysfunction. Consequently, the term PD “dysgraphia” has been proposed to describe this more comprehensive set of handwriting impairments. This shift in terminology is not merely semantic; it reflects significant theoretical and practical implications. Theoretically, it recognizes that motor symptoms typical of PD, such as bradykinesia, tremor, rigidity, akinesia, and freezing, can disrupt handwriting kinematics without necessarily affecting size. Practically, it underscores the value of dynamic and kinematic variables as more sensitive biomarkers for diagnosis, disease monitoring, and therapeutic evaluation [24].

Currently, PD diagnosis and monitoring are primarily based on clinical evaluation of motor symptoms, including bradykinesia, resting tremor, and rigidity [32]. However, in vivo diagnostic criteria still carry an error rate of around 20%, largely due to the lack of objective biomarkers [33,34].

Motion analysis, divided into kinetic (forces and their effects) and kinematic (motion in terms of position, velocity, and acceleration) approaches [35], offers a promising solution. Wearable motion sensors have already demonstrated potential in objectively assessing PD motor symptoms such as bradykinesia [36], rigidity [36,37], tremor [38], and gait and balance impairments [39]. They also offer opportunities for more precise symptom tracking and treatment management [40].

Kinematic gait studies have consistently shown increased stride-to-stride variability, reduced stride length, and impaired rhythmicity in PD patients compared to healthy individuals. Although stride duration itself may remain unchanged, variability in temporal parameters is notably higher in PD [41]. On the kinetic side, analysis of ground reaction forces (GRFs) during gait has revealed characteristic abnormalities. In early PD, a delayed heel strike and premature forefoot loading have been observed, suggesting potential early markers of disease that may be independent of stage or treatment [35,42].

Handwriting, like gait, is a complex motor activity requiring coordination across sensory, cognitive, and motor domains [43]. Legible handwriting depends on the smooth coordination of hand, arm, and finger movements, continuously guided by visual and haptic feedback [44]. In PD, handwriting becomes a consciously controlled, visually dependent task, often executed more slowly than in healthy individuals. Alterations in the kinematics of handwriting have recently been proposed as valuable digital biomarkers for PD, with growing evidence supporting their role in early diagnosis and disease progression monitoring [45].

### 2.3. Traditional Methods for Analyzing Handwriting in PD

Traditional approaches to handwriting analysis in PD rely on various manual and observational techniques to assess handwriting impairments. These methods typically involve pen-and-paper tasks evaluated through visual inspection or standardized scoring systems. Assessments of handwriting in Parkinson’s disease involve various writing tasks [46], including graphical elements such as “e,” “m,” “q,” and “s” [47,48], as well as circles or spirals [48,49], individual letters [50,51], words [49], and complete sentences or text [50]. A significant limitation is the absence of methods for continuous writing assessment, with existing approaches mainly relying on the extraction of individual letters from sentences through unsupervised clustering [31] or distinguishing between pen-up and pen-down movements [51]. These tasks can be performed on different media, such as paper or tablets [49,50], although subjects generally prefer paper [51]. Additionally, handwriting tasks can be recorded using a camera [49].

The visual characteristics of handwriting are primarily analyzed in terms of size [48,51,52] and slant [52]. Fine motor skills, motor control, coordination, and cognitive functions involved in handwriting tasks are evaluated using kinematic features such as position and coordinates, velocity, acceleration [51], and fluency or jerk [47,49]. However, as noted in [46], kinematic features are highly dependent on the specific task.

Dynamic aspects of handwriting, which reflect motor control and cognitive processes, are assessed through parameters such as duration [52], altitude and azimuth, tilt, and pressure. Muscle activity during writing can also be analyzed using surface EMG [48]. Additionally, in-air movements represent a relevant factor in handwriting evaluation [52].

Gallo-Aristizabal et al. (2025) highlighted that while most handwriting studies in Parkinson’s disease focus on geometric figures and spiral drawings, tasks involving the writing of numbers, letters, words, or sentences are more representative of daily life and are often preferred by clinicians during assessments [53]. These semantic writing tasks may also involve a higher cognitive load, offering insights into motor planning and coordination deficits commonly observed in PD [54]. The study further examined dynamic signals from handwriting using multiple feature domains, including kinematic [55,56], spectro-temporal [57], and non-linear [58], and emphasized that time-dependent features, such as pressure and kinematics, captured through digital tablets outperform image-based features in distinguishing PD patients from healthy controls.

Researchers have long used these assessments to identify characteristic handwriting deficits in PD, such as micrographia, inconsistent letter sizing, altered writing slant, and reduced movement fluency [59,60].

While these traditional methods provide valuable clinical insights, they have several limitations. They are often subjective, time-consuming, and prone to inter-rater variability, affecting reliability and consistency [61]. Moreover, manual handwriting assessments may fail to capture dynamic aspects of movement, such as velocity, acceleration, and pressure fluctuations, which are critical for understanding motor control impairments in PD [62]. Despite these challenges, conventional handwriting analysis remains a widely used approach, especially in clinical settings where technological alternatives may not be readily available.

## 3. Artificial Intelligence and Its Role in Healthcare

### 3.1. Definition and Core Concepts of AI

AI represents one of the most expanding and revolutionary forces in modern medicine. It refers to the ability of machines and computational systems of simulating human cognitive functions, such as learning, reasoning, and problem-solving [63]. While the term itself was coined by John McCarthy in 1956, the concept of machines performing human-like tasks can be traced back to Alan Turing’s foundational work in computing and the famous Turing test for machine intelligence [64,65]. In healthcare, AI has become increasingly integrated into clinical and non-clinical environments, enabling faster and more accurate diagnosis and decision-making [66], optimizing treatment strategies for patients across diverse specialties [65,67], and aiding in studying disease progression, evaluating readmission and complication risks, and foreseeing mortality prediction [68].

The evolution of AI in medicine can be conceptualized through three historical epochs. The first epoch involved symbolic AI and probabilistic models, where medical knowledge was encoded into rule-based systems. These systems were valuable but often limited in capability with real-world situations. The second epoch, defined by the authors as AI 2.0 and starting from 2011, was marked by the rise of ML and deep learning (DL) technologies [69]. This phase enabled breakthroughs in image recognition, diagnostics, and outcome forecasting. The most recent phase, AI 3.0, features foundation models and generative AI, such as large language models, which are capable of multitasking, contextual understanding, and text generation without retraining—offering a new paradigm of versatility and interactive use in clinical settings [69].

Among the most widely used subfields of AI in healthcare are ML, DL, and pattern recognition.

### 3.2. Machine Learning, Deep Learning, and Pattern Recognition

As first defined by Arthur Samuel in 1959, ML is a subfield of AI focusing on allowing computers to learn from data and improve their performance over time without being explicitly programmed. ML empowers machines to acquire knowledge from experience, reducing the need for manual programming. Tom M. Mitchell later formalized this concept, writing “a computer program is said to learn from experience E with respect to some class of tasks T and performance measure P, if its performance at tasks in T, as measured by P, improves with experience E.” At the core of ML are algorithms, which are mathematical procedures that transform input variables into output results. These are designed to adapt and optimize based on the information they process [70]. This adaptability, akin to human learning, allows ML systems to improve over time, becoming more accurate as they encounter more data. These algorithms can infer relationships within data, whether simple and linear or highly complex. When the associations in a dataset are too intricate to be captured by conventional statistical models, such as linear regression, ML provides an alternative by using flexible, data-driven models that can uncover previously hidden patterns [71]. The learning process typically follows three essential steps, starting from a decision process, where a prediction is posed on available data; then, the prediction is evaluated against known outcomes using an error function, and eventually adjustments are made to minimize future errors. This iterative feedback loop enables the model to refine its internal parameters, increasing its predictive performance over time.

Machine learning encompasses a broad range of methodologies, broadly categorized into supervised, unsupervised, semi-supervised, and reinforcement learning [72,73].

ML models are built upon statistical principles and are widely classified into parametric and non-parametric, each suited to different data structures and analytical purposes. Parametric models, such as linear and logistic regression, rely on fixed-size parameter sets and make strong assumptions about the underlying data distribution, enabling efficient learning when the data aligns with these assumptions. Conversely, non-parametric models, including decision trees and support vector machines (SVMs), offer greater flexibility by making fewer assumptions about data structure, although they often require larger datasets to achieve high performance. Decision trees provide an intuitive, hierarchical approach to classification and regression, while random forests, as ensembles of decision trees, increase predictive accuracy through aggregation. SVMs are powerful tools for complex classification tasks, particularly in high-dimensional spaces where linear boundaries are insufficient [74].

**Figure 1:**
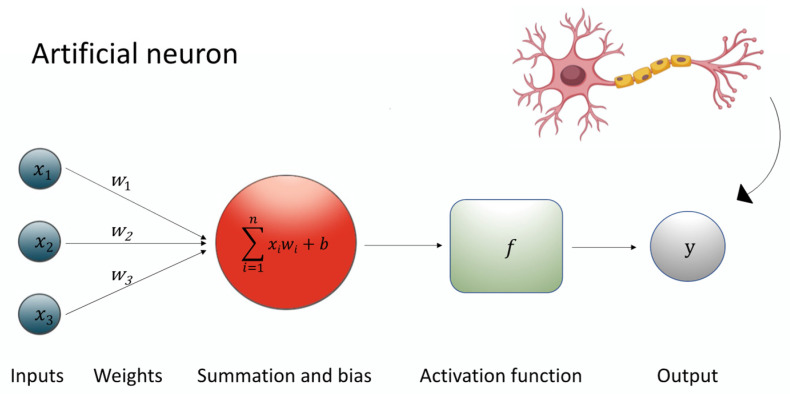
The figure shows the equation underlying the artificial neuron.

A specialized subset of ML, deep learning (DL), utilizes leverages of multi-layered neural networks to automatically learn hierarchical representations of data [75]. As seen in Figure 1, at the heart of this architecture lies the artificial neuron, a mathematical unit that processes inputs (*Xi*) through associated weights (*w*), adds a bias term (*b*), applies a summation function (*∑*), and then passes the result through a non-linear activation function (*f*) to produce an output signal (*y*). This process can be expressed through the equation: *y* = *f* (*∑wi* × *Xi* + *b*) [76].

**Figure 2:**
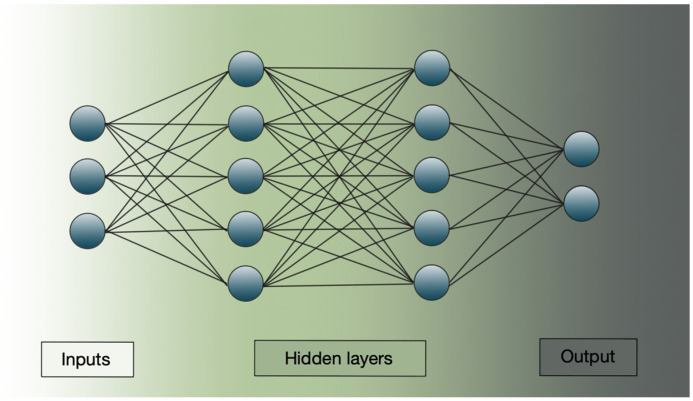
Schematic representation of a deep learning structure. The image shows input layers, hidden layers, and the output result.

By stacking multiple layers of such neurons—known as hidden layers—deep learning models are able to transform inputs into abstract and complex representations. These hidden layers form the “depth” in deep neural networks, allowing them to approximate highly non-linear mappings between inputs and outputs (Figure 2) [77].

DL techniques are generally classified into deep networks for supervised or discriminative learning (mainly including Multi-Layer Perceptron (MLP), Convolutional Neural Networks (CNNs) and Recurrent Neural Networks (RNNs)), deep networks for unsupervised or generative learning (e.g., Generative Adversarial Network (GAN) and Autoencoder (AE)), and hybrid models or other approaches such as deep transfer learning (DTL) and deep reinforcement learning (DRL). Each network is tailored to specific learning paradigms. Discriminative models are used in supervised tasks and focus on classification accuracy by directly learning the likelihood of a certain class given an input, effectively “discriminating” between classes without modeling the full underlying structure or distribution of the input data. Generative models, by contrast, aim to learn the joint distribution of input data and their labels, enabling tasks such as feature learning, representation learning, or data synthesis—often without requiring labeled outputs. These models can also enhance supervised learning by providing richer, more informative input representations. Hybrid models combine both approaches, leveraging labeled and unlabeled data to improve generalization and performance, particularly in complex or insufficient-data scenarios [76]. Like traditional ML, DL models follow a structured workflow comprising data understanding and preprocessing, model building and training, and validation and interpretation, with the key distinction that DL automates the feature extraction process [77,78].

Pattern recognition refers to the automated process of identifying regularities or patterns in raw data and making decisions or classifications based on these identified categories. As defined by Duda et al., it is “the act of taking in raw data and taking an action based on the “category” of the pattern” [79]. In essence, pattern recognition involves analyzing the properties of objects and assigning them to predefined classes or categories, often serving as a foundational task in machine learning, image processing, and data analysis [80].

### 3.3. AI Applications in Neurology and Movement Disorders

AI applications have spread widely across clinical medicine. Diagnostic tools using AI have achieved impressive results in radiology, cardiology, dermatology, oncology, and more, often matching or surpassing human expertise in accuracy [68]. ML has been used for detecting diabetic retinopathy from retinal images [69,78], to analyze tumor histology [78], to predict in-hospital mortality in neonates with clinically suspected sepsis [81], forecast outcomes after traumatic brain injury, and stratify patients based on risk in critical care settings [82]. For instance, in their review, Baloglu et al. [71] list some of the uses of ML algorithms in pediatric care, such as predicting favorable versus unfavorable outcomes at 6 months post traumatic brain injury.

DL has been effectively applied to disease diagnosis, risk prediction, medical imaging, and COVID-19 detection, often surpassing conventional models in performance. Its capacity to process high-dimensional, unstructured data makes it a foundational technology in the development of intelligent healthcare systems and personalized medicine [76].

AI-driven clinical decision support systems are now assisting with treatment planning, prognosis prediction, early detection of disease, risk stratification, and the personalization of care plans [73]. Such tools contribute not only to efficiency but also to improved outcomes and reduced costs, enabling physicians to focus more on direct patient care [67]. The integration of AI into healthcare also introduces complex ethical concerns. A critical issue is algorithmic bias, where AI systems trained on unrepresentative or biased data can reproduce and even amplify existing health disparities. Another major concern is the explainability of AI outcomes, particularly for complex DL systems, which often function as “black boxes” [74]. Explainable AI (XAI) seeks to bridge this gap, aiming to make AI decisions transparent and understandable to users, an essential feature in clinical settings. Recent studies in the scientific literature have highlighted the multifaceted impact of XAI in clinical contexts [83,84]. Gombolay et al. [83] explored how different XAI techniques affect clinical decision-making in neurology, particularly when used as part of AI-based decision support systems. The effect of XAI is not universally positive but varies significantly depending on the physician’s level of expertise and their perception of the system’s explanations. More experienced neurologists performed worse when faced with highly explainable XAI, suggesting that it may interfere with the intuitive, model-based decision-making processes that experts typically rely on. In contrast, less experienced physicians tended to benefit from XAI, especially from its intuitive and easy-to-follow explanations. Furthermore, Rosenbacke et al. [84] show that explainability can both build and erode confidence, depending on clarity and relevance. These findings suggest that current XAI methods often fail to make complex models truly transparent, emphasizing the need for adaptive, tailored strategies to clinical reasoning. Moreover, the deployment of AI tools must be guided by principles of fairness, justice, accountability, and respect for patient autonomy. Human oversight remains crucial to maintain trust, ensure ethical use, and safeguard against inappropriate automation in morally complex clinical decisions [85,86]. In response to the growing role of AI in critical sectors, the European Union signed the Artificial Intelligence Act in June 2024 [87], establishing the first comprehensive legal framework for AI regulation. The Act imposes specific safety, transparency, and oversight requirements, especially for high-risk systems such as AI-powered medical devices, aligning them with the EU Medical Devices Regulation through mandatory third-party conformity assessments [88].

The role of AI in neurology and movement disorders is especially promising. In the field of neuro-oncology, AI is being used to support the management of gliomas, which are the most common and aggressive tumors of the central nervous system (CNS). Luo et al. [89] explored the emerging application of ML and DL models across various stages of glioma care, including tumor segmentation, diagnosis, grading, outcome prediction, and treatment planning. Although the integration of AI into routine clinical practice remains limited, the authors express an optimistic position regarding its potential. A noteworthy example is provided by Shen et al. [90], who demonstrated the successful real-time intraoperative diagnosis of gliomas using deep CNNs in combination with near-infrared II (NIR-II) fluorescence imaging. This approach enabled accurate tumor identification during surgery, representing a significant advancement in AI-assisted neurosurgical decision-making [90].

In the context of cerebrovascular diseases, instead, AI is increasingly employed to support prognostic modeling and personalized therapy planning in post-stroke rehabilitation. Campagnini et al. (2022) [91] conducted a comprehensive review of ML applications aimed at predicting motor recovery, highlighting that while current models vary in quality, they show significant potential. Linear and logistic regression were the most commonly used algorithms, favored for their interpretability and clinical relevance. Despite limitations such as small sample sizes, limited feature reporting, and heterogeneous outcome measures, AI-driven models hold promise for optimizing rehabilitation strategies tailored to individual patient profiles [91]. Beyond these applications, AI has also been implemented in assessing complex movement disorders. In their systematic review and diagnostic meta-analysis, Ghaderi et al. [92] studied the application of ML to MRI data to identify affected brain regions in motor-impaired patients, enhancing diagnostic clarity. Balachandar et al. [93] explored the use of DL models with deep brain stimulation data to monitor sleep patterns in movement disorder patients, demonstrating improved clinical insight through real-time signal analysis. In their systematic review, Vizcarra et al. [94] summarized the existing literature regarding the use of AI for diagnosis and quantitative phenotyping of hyperkinetic movement disorders, including ataxia, chorea, dystonia, myoclonus, tics, and tremor. Among the 55 included studies, the most used ML method was SVM, while CNNs were the most frequent DL approach. Diagnostic outcomes were a key focus: 38 studies dealt with disease diagnosis, reporting accuracies ranging widely from 56% to 100%, depending on the disorder and dataset used. On the quantitative phenotyping side, 23 studies examined how well AI could mirror or predict clinical severity ratings. Only 10 of these reported statistical correlation values between AI predictions and clinical ratings, ranging from 0.54 to excellent (0.99). Despite promising results, the authors noted major limitations in study quality and transparency. While internal validation was widely used, only three studies employed external validation, a critical step for assessing real-world utility. The authors argue for stricter adoption of AI-specific research guidelines, like STARD-AI and TRIPOD-AI, to improve reporting transparency and model interpretability [94].

Eventually, a growing area of interest is the application of AI in PD. For instance, Purk et al. [95] introduced a tablet-based AI platform that integrates motor drawing features with symptom questionnaires, achieving over 90% diagnostic accuracy, while Huo et al. [96] developed a graph-based DL model that captures subtle gait disturbances, significantly improving early PD detection. Collectively, these findings affirm the transformative role of AI across the neurological spectrum. From brain tumors to stroke and movement disorders, and especially in PD, AI is advancing early diagnosis, refining clinical decision-making, and supporting more personalized patient care. In the following subsections, we specifically discuss AI-based handwriting analysis and clinical application in PD.

## 4. AI-Based Handwriting Analysis in Parkinson’s Disease

### 4.1. Data Collection Methods: Paper, Digital Tablets, and Smartpens

Handwriting analysis has become a key method for the early detection and monitoring of PD, supported by advances in AI [97].

Data can be collected using both offline methods, such as traditional pen-and-paper tasks, and online methods, which involve digital tools like tablets and smartpens. Offline tasks, writing letters, spirals, or sentences, are often more familiar and natural for older adults, but they lack temporal and dynamic information unless later digitized and analyzed [98].

In contrast, online handwriting offers real-time, high-resolution capture of handwriting dynamics, including pressure, velocity, acceleration, and pen inclination [99]. Smartpens and digital tablets allow for unobtrusive, naturalistic assessments, with some setups incorporating flexible sensing arrays that avoid altering writing behavior. These technologies ensure that subtle motor symptoms, often invisible in static samples, are recorded with rich temporal and spatial detail, providing a robust basis for AI-based analysis.

Recent studies also emphasize the potential of digital platforms for early screening and remote monitoring, enabling continuous assessment outside clinical settings and early detection of motor deterioration [99,100]. Data collected passively at home could help identify symptom changes before they become clinically apparent.

A study by Walker and colleagues evaluated the Manus platform, a portable system where older adults completed standardized writing and drawing tasks using a digital pen on a flat screen. Movement time and velocity were used to assess bradykinesia, while writing size was analyzed for micrographia. Rest tremor was examined via frequency analysis during a resting recording. The system demonstrated strong diagnostic performance, distinguishing PD patients from controls with 90% sensitivity and 80% specificity. PD patients, who were in early disease stages, showed slower movements and smaller handwriting. Tremor detection also aligned with clinical diagnoses [101].

Overall, both paper-based and digital methods remain relevant. While traditional handwriting tasks are clinically well-established and easy to administer, digital tools greatly enhance the quality and quantity of information collected, making them especially valuable for AI-driven analysis and long-term monitoring.

### 4.2. Key Features Extracted: Spatial, Temporal, and Kinematic Parameters

In the analysis of handwriting for PD, feature extraction primarily targets the spatial, temporal, and kinematic domains. Among spatial features, micrographia is a well-known hallmark of PD. Temporal features focus on writing duration, stroke timing, and fluency, which are directly affected by bradykinesia and other motor impairments. Kinematic features capture aspects such as velocity, acceleration, jerk (a measure of movement smoothness), and pressure variations [62].

Studies show that handwriting impairments extend beyond micrographia to broader dysgraphia, involving disturbances in fluency, coordination, and motor planning. Importantly, dynamic kinematic variables (velocity, acceleration, fluency) outperform static measures (like size alone) in reliably distinguishing PD patients from healthy controls [102]. Rios-Urrego et al. [58] conducted a detailed comparative study, extracting kinematic, geometrical, and non-linear features from handwriting tasks (spirals, meanders, circles). Their findings indicated that kinematic features consistently achieved the highest classification performance, with accuracies above 93% when distinguishing PD patients from healthy subjects. This reinforces that dynamic handwriting behavior provides a more sensitive biomarker than static characteristics alone. Handcrafted features commonly used in handwriting analysis can be divided into conventional and advanced features [54], which are illustrated in Table 1.

Advanced features encompass more complex representations, such as entropy measures, signal-to-noise ratio (SNR), empirical mode decomposition (EMD) [54], cepstrum [103], and sigma-lognormal models [104].

Emerging evidence increasingly supports handwriting kinematics as early digital biomarkers for PD diagnosis and prediction. Subtle kinematic alterations in handwriting can precede clinical diagnosis, opening new possibilities for preventive interventions.

### 4.3. Algorithms and Techniques: Supervised Learning Models and Deep Learning Architectures (CNNs, LSTMs)

Following feature extraction, ML models are deployed for classification and diagnosis. Recently, DL techniques, particularly CNNs and Long Short-Term Memory networks (LSTMs), have revolutionized handwriting analysis. CNNs are excellent for learning spatial hierarchies in image-based handwriting data, while LSTMs handle the temporal sequencing of handwriting motions.

However, the necessity of manual design and development is still a severe limitation. Recent advancements in artificial neural networks offer new possibilities for automated feature extraction. By utilizing transfer learning, pre-trained CNNs can be advantageously used to extract features and, as such, provide an alternative solution in place of tedious and time-consuming manual feature design. This approach has already been used not only for handwriting processing [105] but also in several other domains [106]. Nevertheless, in the area of handwriting processing, one apparent limitation of CNN feature extraction is that it utilizes only image data, and as such, it is limited only to offline handwriting processing. However, there have recently been some promising attempts to employ recurrent neural networks for the classification of handwriting signals [107].

Various parametrization techniques for offline and online handwriting have been developed. However, a major limitation of the current state of affairs is that these techniques are treated separately most of the time. Zhang et al. implemented a ResNet-18 CNN architecture for classifying Parkinsonian handwriting with high accuracy, using pressure maps as input [108]. Likewise, Li et al. demonstrated that deep neural networks applied to motion data can robustly assess Parkinson’s disease severity [109]. Benredjem et al. explored deep learning frameworks combining handwriting samples and kinematic dynamics, achieving notable prediction rates for early-stage PD [110]. Their research highlights that multimodal approaches, merging pressure, speed, and acceleration data streams, significantly outperform traditional static image-based methods.

Importantly, AI methods are not only able to detect PD but also predict the risk of conversion from a prodromal stage to manifest PD by analyzing early handwriting anomalies. This predictive capability could revolutionize screening programs and enable earlier therapeutic interventions.

In a 2025 study, Gallo-Aristizabal and colleagues compared image-based and signal-based approaches to analyze online handwriting signals collected from PD patients and healthy controls. The study explored three methods: one using images derived from handwriting to feed a CNN and two using time-dependent features, specifically, pressure and kinematic signals, modeled through statistical functionals and Gaussian Mixture Models (GMMs). The results showed that dynamic, signal-based analyses significantly outperformed the image-based approach, especially when using pressure features with statistical functionals, suggesting that vertical control during handwriting is more impaired in PD patients than horizontal movement [53].

The CNN-based image analysis achieved better performance when more layers were fine-tuned, but it still did not match the accuracy of the dynamic methods. Among signal-based methods, the statistical functional approach yielded higher accuracy than GMMs, particularly for pressure signals, and had the added advantage of being more interpretable and computationally efficient for clinical use. Interestingly, combining pressure and kinematic features did not enhance performance, and kinematic features performed similarly with both statistical models. The study concluded that pressure-based dynamic features are the most informative for distinguishing PD patients from controls and suggested future work should include a broader range of writing tasks and larger datasets to enable more advanced modeling approaches, such as recurrent neural networks [53].

A 2024 study by Wang and collaborators revealed that handwriting can serve as a highly sensitive marker of motor impairments, such as tremor, rigidity, and bradykinesia, offering a promising avenue for early PD detection. The researchers introduced a lightweight and efficient neural architecture, LSTM-CNN, designed to analyze local time-dependent handwriting segments rather than holistic samples or manually crafted features [111].

The handwriting signals, captured via digital tablets or smart pens, were segmented through a sliding window approach. Each segment was then analyzed by a hybrid 1D neural network, combining LSTM units to capture temporal dependencies and 1D CNNs to extract spatial patterns [112,113]. The proposed method demonstrated strong diagnostic capability across different datasets, accurately distinguishing between individuals with PD and healthy controls. Its performance remained consistently high, showing strong sensitivity and specificity across evaluations. Importantly, the model maintained excellent computational efficiency, with a very small number of parameters and minimal processing time per sample. This allows for fast, near-real-time diagnosis, making it practical for clinical or even remote applications. Further analysis showed that the spatial trajectory of handwriting, specifically the x and y coordinates, was the most informative for detecting signs of Parkinson’s. Additionally, a majority voting mechanism, which aggregates predictions from multiple segments, was used to increase the reliability of the final diagnosis. Compared to traditional ML models and other DL architectures, the LSTM-CNN model showed superior performance both in accuracy and efficiency.

### 4.4. Hybrid Approaches Combining Signal Processing and AI

Hybrid approaches in the analysis of handwriting for PD involve the integration of different computational strategies to enhance diagnostic accuracy. Traditionally, this refers to the combination of signal processing techniques, used to extract meaningful features from handwriting movements such as velocity, pressure, and acceleration, with AI models capable of learning patterns from these features and classifying them effectively.

However, a broader interpretation of hybrid modeling has emerged, particularly in image-based handwriting analysis, where deep learning networks are used to automatically extract features from raw input data (such as digitized spiral drawings), and the extracted features are then passed to separate ML or DL classifiers for final prediction. This structure benefits from deep networks’ ability to capture complex, hierarchical representations of input data while leveraging the classification strength of traditional models such as SVMs or fully connected neural networks.

In the study by Varalakshmi et al. [114], this type of hybrid approach is applied to spiral drawings, which serve as a useful biomarker for PD due to their ability to reflect fine motor impairments. Given the small size of the initial dataset, the authors use data augmentation techniques to artificially expand the training set and improve model generalization. They then evaluate a variety of models, including ML algorithms, DL architectures, pretrained convolutional networks, and hybrid combinations.

**Figure 3:**
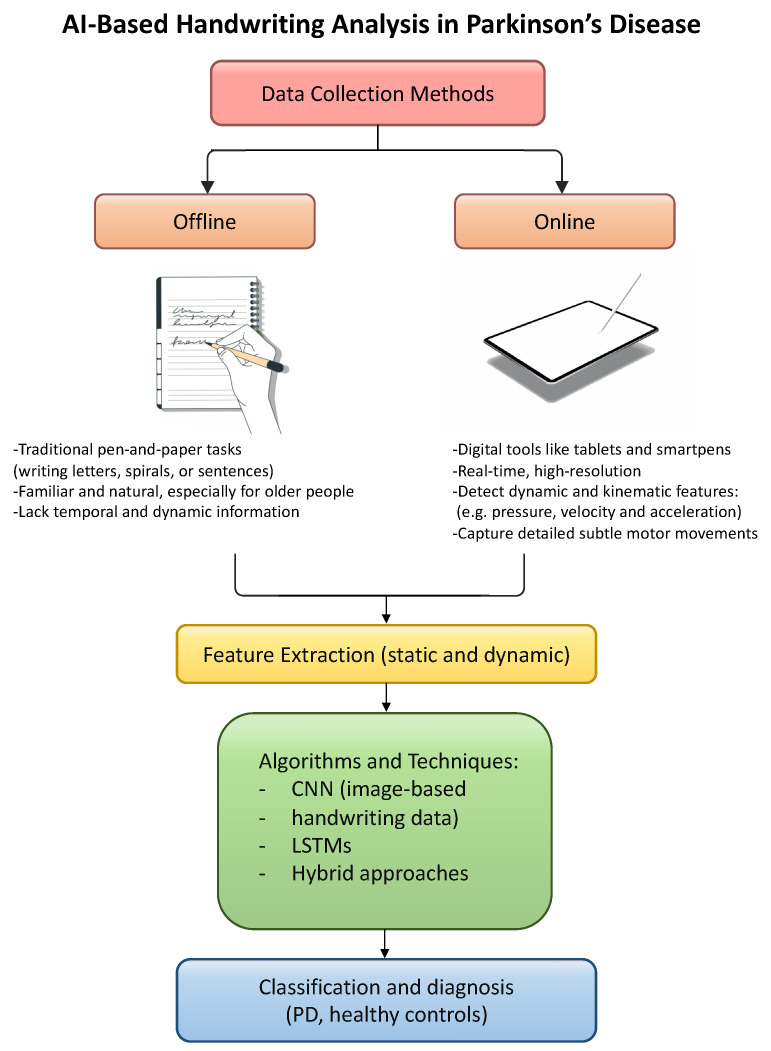
Schematic representation of AI-based handwriting analysis in PD.

Their findings show that hybrid models, particularly those that combine a pretrained deep learning model, like RESNET50, with a ML classifier such as SVM, are especially effective. These models outperform standard approaches by extracting more informative features and achieving higher classification performance. This suggests that hybrid strategies, not only those combining signal processing with AI but also those integrating different AI paradigms, can significantly enhance the early detection of PD through handwriting analysis. The workflow for detecting Parkinson’s disease through handwriting analysis with AI algorithms is shown in Figure 3.

## 5. Clinical Applications and Research Findings

### 5.1. Early Diagnosis and Screening

Although there is currently no cure for PD, several clinical methods have been developed to assess the severity and progression of the condition. These tools are essential in improving the quality of life for individuals living with PD [115]. As such, early prediction and diagnosis are critical for effective disease management.

It is widely recognized that Parkinson’s may begin years before the onset of motor symptoms, with early indicators including a diminished sense of smell, disrupted sleep, constipation, tremors, and bradykinesia. Additionally, approximately 90% of patients with PD experience vocal impairments [116]. For this reason, researchers are increasingly focused on identifying these non-motor symptoms as potential early markers of the disease, aiming to intervene at earlier stages and possibly slow its progression [117].

A study comparing traditional clinical diagnostic methods with post-mortem neuropathological confirmation found that clinical diagnosis showed a sensitivity of 88% but a lower specificity of 68% [118]. More concerning, early-stage diagnosis had significant limitations, correctly identifying only 26% of patients who were untreated or showed unclear responses and 53% of those responding to medication [1].

Because PD is a movement disorder, diagnosis often occurs later in life, after patients have unknowingly lived with the disease for some time. This is largely due to diagnosis relying on the observation of motor difficulties such as tremors and dystonia-like symptoms [119]. Moreover, most cases of Parkinson’s and other dementias are diagnosed at a stage when significant neurological deterioration has already occurred [120]. As a result, patients often become aware of their symptoms only when they are already experiencing persistent and debilitating tremors or other motor issues, which are generally considered late-stage signs of the disease [121].

Given these challenges, the development of artificial intelligence tools capable of detecting biomarkers associated with PD at an earlier stage is vital [122]. The improved predictive accuracy observed in AI-driven approaches can be attributed to their ability to detect subtle changes that traditional methods often fail to identify. Additionally, AI allows for the simultaneous analysis of multiple handwriting features. In recent years, a wide range of AI technologies, including artificial neural networks and ML models, have emerged to assess both the severity and progression of neurodegenerative diseases such as PD, Alzheimer’s, and dystonia. Various studies have applied ML to distinguish PD from other neurological conditions, although one of the main challenges lies in feature engineering, which can be resource-intensive.

To address this, researchers have increasingly turned to DL techniques like CNNs and RNNs, which offer automated feature extraction, enhanced pattern recognition, and high diagnostic accuracy [123]. These approaches have been successfully used to diagnose PD early, especially when combined with wearable sensors and data-driven algorithms, achieving accuracy rates above 90% in distinguishing patients from healthy individuals [124]. Additionally, symptom severity prediction has shown promising results through the integration of diverse sensor inputs and machine learning models.

However, the implementation of AI-based diagnostics still faces challenges, such as limited scientific validation, lack of clinical testing, and inadequate big data infrastructures [125]. To ensure the reliability and precision of AI tools used for PD diagnosis, it is essential to reduce algorithmic bias and validate models using well-structured and sufficiently large datasets.

### 5.2. Disease Progression Monitoring

Common handwriting-related impairments in PD patients include micrographia and dysgraphia, which refers to the difficulty in executing the precise and coordinated motor movements necessary for writing [126]. Currently, the progression of motor symptoms in PD is primarily assessed using the Movement Disorder Society-Unified Parkinson’s Disease Rating Scale, part III (MDS-UPDRS-III), which is administered by trained neurologists [127]. This clinical tool includes several motor assessments that are directly or indirectly associated with handwriting, such as finger tapping, hand tremor, and rigidity. However, this process is often demanding, posing a burden in terms of time, cost, and effort for patients, caregivers, and healthcare systems [128].

In this context, automated handwriting analysis is emerging as a promising complementary method to support both diagnosis and monitoring of PD. There is a growing interest in the research community in using computational approaches to objectively assess handwriting performance in PD patients. Most studies focus on online handwriting, using digital tablets that record dynamic data during the act of writing, specifically when the pen is in contact with the surface (on-surface data).

Aligned with this technological direction, it has been proposed that computerized analysis of handwriting provides a simple, non-invasive, and effective tool that can assist clinicians in diagnosing PD and tracking the progression of dysgraphia in affected individuals [126]. Altogether, accumulating evidence supports the notion that PD-related dysgraphia may serve as a reliable physiological biomarker for the early detection and ongoing monitoring of Parkinson’s disease [126,129].

### 5.3. Treatment Response Evaluation

While levodopa remains the cornerstone of pharmacological treatment for PD, motor rehabilitation represents a crucial non-pharmacological adjunct [130]. Several studies have demonstrated that motor learning in PD can be enhanced through cueing and feedback strategies [131,132]. Cues, whether visual or auditory, act as external triggers for initiating movement, while feedback provides additional external information to compensate for impaired internal sensory pathways, thereby supporting the motor learning process.

Evidence shows that short-term training using visual cues and feedback is particularly effective in improving gait in PD [133,134], with examples including treadmill walking and cycling exercises incorporating visual stimuli [135,136]. More recently, visual cue-based rehabilitation has also been shown to improve upper limb tasks [137].

In the context of handwriting rehabilitation, repetitive letter formation may reinforce sensory feedback loops, which in turn enhance corticomotor excitation and promote neuroplasticity. One of the most common handwriting impairments in PD is micrographia, which is thought to result from poor coordination between wrist and finger movements, as well as wrist stiffness, although its precise mechanisms remain unclear [138]. Nonetheless, several studies have shown the benefits of targeted handwriting training. Ziliotto et al., for example, reported improved writing performance following a 10-week rehabilitation program [139]. Similarly, a six-week intensive amplitude training protocol led to improvements in writing amplitude and its variability, indicating a role for motor learning consolidation in PD [140]. The study by Nackaerts et al. further supports the efficacy of amplitude-based training. The authors found that, beyond previously reported improvements in handwriting size, intensive amplitude training also led to increased movement duration and decreased writing fluency across a range of task conditions. In contrast, the placebo group showed no improvement in writing amplitude, though it did exhibit shorter movement duration and improved fluency in automatization tasks [140].

These findings suggest a trade-off within the motor control system of individuals with PD, wherein prioritizing one movement parameter, such as amplitude, may compromise others, including speed and fluency [141]. Such compensatory effects may be influenced by cognitive reserve and disease severity, particularly in more advanced patients [142].

Interestingly, some improvements in fluency and speed were observed during the retention phase following training [140], despite the absence of continued practice. This underscores the importance of focusing rehabilitation on a single, functionally relevant parameter, such as amplitude, to enhance handwriting legibility, while progressively adapting the intervention to accommodate the individual’s motor capacity.

Thus, artificial intelligence tools, previously discussed as promising instruments for the diagnosis and monitoring of Parkinson’s disease through handwriting analysis, may also hold value in objectively evaluating treatment response and optimizing individualized rehabilitation strategies.

## 6. Advantages and Limitations of AI-Based Handwriting Analysis

### 6.1. Non-Invasive and Cost-Effective Monitoring

The integration of AI and ML into healthcare has sparked growing interest due to their transformative potential in clinical environments. Among its various applications, AI-based handwriting analysis represents a promising approach for non-invasive and cost-effective patient monitoring. The reviewed literature identifies a range of advantages as well as critical limitations that must be considered when implementing these technologies.

AI tools offer significant benefits, including improved productivity, enhanced performance compared to traditional methods, optimized hospital administration, attraction of skilled professionals, cost savings, and a reduction in clinician workload. Moreover, such technologies have been linked to better patient care outcomes, systemic improvements, and a reduction in healthcare disparities [143,144,145,146,147].

A frequently emphasized benefit is the enhancement of workflow efficiency. AI can automate routine administrative tasks such as paperwork and documentation, thereby allowing clinicians to devote more time to direct patient care [143,144,145,146,147]. However, some studies suggest that while initial productivity gains are notable, their long-term sustainability remains uncertain [147]. Over time, the time saved may be reabsorbed into other tasks, diminishing the lasting impact unless there is intentional workflow redesign and continuous user engagement [146].

Despite these advantages, the implementation of AI-based systems, such as those for handwriting analysis, also involves significant challenges. Upfront investments in infrastructure and technology are often required, presenting economic barriers, particularly for resource-constrained institutions [148]. Additionally, healthcare providers must carefully weigh these costs against the anticipated long-term benefits.

Another concern is the potential for widening inequalities. Institutions with greater resources and access to scarce AI expertise may adopt these innovations earlier, potentially deepening existing healthcare disparities [149]. Furthermore, challenges related to government regulation, policy compliance, technological limitations, organizational readiness, social acceptance, and ethical considerations must be addressed to ensure the successful and equitable adoption of AI in clinical settings.

While economic and social barriers remain significant, several collaborative and systemic strategies are emerging to address them. Low-cost digital tablets and open-source handwriting analysis software are being developed to reduce financial costs. In order to make those tools accessible, it is necessary to create multi-institutional collaborations and to enable the transition from private to public. Moreover, the involvement of clinicians and patients in the design of AI systems through participatory research and user-centered design may help improve usability, aligning technological solutions with real-world needs.

### 6.2. Challenges in Data Quality and Standardization

Despite the promising potential of AI-based handwriting analysis for non-invasive and cost-effective patient monitoring, several data-related challenges limit its broader adoption and effectiveness. A widely cited concern is the risk of embedding and amplifying biases within AI systems [143,144,145,146,148]. These biases, often rooted in unrepresentative or flawed training data, can result in inequitable healthcare outcomes, disproportionately affecting marginalized populations, such as racial and ethnic minorities and women [149,150]. Biases may emerge from skewed data composition, incomplete labeling, or contextual factors that are not accounted for during algorithm development.

To address bias in AI handwriting analysis, it is essential to conduct a multidisciplinary approach, engaging different experts of the field, such as data scientists, engineers, developers, healthcare professionals, and ethicists throughout all the model life cycle. [151]. Bias can be mitigated by ensuring diverse and representative handwriting datasets, particularly taking into consideration differences in age, gender, ethnic group, and dominant hand. Oversampling techniques, such as SMOTE, can be used to rebalance underrepresented classes, as demonstrated in handwriting-based models for Parkinson’s detection [152]. Stratified model evaluation and fairness metrics should be routinely employed to identify performance disparities. Additionally, bias must be addressed continuously through improving governance structures and avoiding institutional oversight, incorporating ethical guidelines and transparent documentation [151].

Data quality, standardization, and accessibility are among the most significant hurdles to implementing AI and ML in healthcare [153]. The healthcare sector often deals with fragmented, highly regulated, and complex datasets that are difficult to harmonize. Without robust and standardized frameworks for data collection, sharing, and annotation, AI systems risk producing unreliable or non-generalizable outputs [154]. Moreover, much of the evidence supporting AI’s clinical use comes from high-income countries with curated datasets, limiting the applicability and scalability of these tools in low- and middle-income settings [155]. While most datasets underpinning targeted therapies primarily derive from Europe and North America, individuals from countries with resource-limited healthcare settings are substantially underrepresented, with a predominance of European ancestry profiles. This geographic and demographic bias is further compounded by infrastructural gaps, as data often remain in non-digital formats, such as paper records, limiting their accessibility and integration into AI-driven systems [156]. These disparities not only restrict the scalability and reliability of AI outputs but also risk perpetuating or exacerbating global health inequities. Training datasets frequently exhibit significant omissions across ethnic groups and among those lacking healthcare access, resulting in models that inadequately serve marginalized populations. Although AI is often heralded as a means to optimize manufacturing and reduce costs, its benefits currently tend to be confined to wealthier countries and populations, particularly given the extremely high costs associated with targeted therapies.

To address these issues, there is an urgent need for the development of ethical data governance models, standardized protocols for data handling, and tools that support responsible data sharing. Providers must also implement ongoing evaluations of AI systems to ensure fairness, transparency, and accountability in clinical decision-making. The emerging concept of “corrective bias” proposes intentionally prioritizing data collection and algorithmic development for underserved groups to help rebalance existing inequities [156]. Recent perspectives highlight that achieving fairness in AI-driven healthcare necessitates not only robust governance but also the adoption of technical and organizational strategies, such as federated learning, model disentanglement, fairness-aware training, and transparent evaluation across relevant subpopulations (e.g., racial, ethnic, socioeconomic), to ensure that AI systems trained on biased or non-representative data do not reinforce or worsen health disparities in real-world applications [157].

Additionally, it is essential that future policies and implementation strategies consciously address existing disparities and actively work to prevent their perpetuation. Rather than unintentionally reinforcing structural inequities through neglect or narrow design, these policies should explicitly promote inclusion, equity, and fair access.

Ultimately, acceptance by both clinicians and patients represents a key challenge in realizing the full benefits of AI systems [143,148]. Physician resistance to changes in traditional workflows and patient skepticism toward algorithm-driven care can significantly hinder adoption [158]. Without stakeholder trust and engagement, even technically robust AI solutions may fail to integrate successfully into clinical practice.

### 6.3. Ethical Considerations and Patient Privacy

The healthcare sector is undergoing a significant transformation driven by technological innovation, particularly in response to longstanding challenges related to scalability, data security, and interoperability. Traditional healthcare infrastructures often struggle with secure data storage, efficient information exchange, and seamless coordination among healthcare providers. In this context, emerging technologies such as blockchain and hybrid DL offer promising solutions to these persistent issues [159].

Blockchain technology, originally developed to support cryptocurrencies like Bitcoin, is increasingly recognized for its potential in healthcare. Its decentralized and immutable architecture enhances data security and transparency. By leveraging cryptographic techniques, consensus algorithms, and smart contracts, blockchain enables the creation of tamper-proof distributed ledgers that support fine-grained access control. This facilitates secure management and sharing of sensitive health data, such as electronic health records (EHRs), medical imaging, and diagnostic reports, while preserving patient ownership and informed consent. Moreover, the decentralized nature of blockchain reduces reliance on intermediaries, lowers administrative costs, and enhances data accessibility, contributing to several Sustainable Development Goals (SDGs) [160].

The integration of blockchain with hybrid DL models further amplifies its utility. While traditional ML often struggles with the heterogeneity and complexity of healthcare data, DL models, such as CNNs and RNNs, excel in recognizing patterns in unstructured data like handwritten medical notes, sensor outputs, and clinical narratives. Hybrid approaches that combine DL with conventional algorithms can offer high accuracy without incurring excessive computational costs.

In their 2023 study, Aitizaz Ali et al. [159] proposed a novel framework titled Blockchain-Powered Healthcare Systems: Enhancing Scalability and Security with Hybrid Deep Learning. Their system integrates a permissioned blockchain with hybrid DL techniques to address key limitations of current healthcare infrastructures. The framework ensures data integrity, privacy, and interoperability, while smart contracts manage consent and automate access control, thereby empowering patients to take ownership of their health data. Advanced techniques such as federated learning and transfer learning further support the real-time analysis of decentralized datasets, enabling timely diagnoses and personalized treatment planning without compromising confidentiality.

Despite these technological advantages, the integration of AI and ML in healthcare, particularly in the analysis of complex data like handwritten medical records, raises important ethical and legal concerns. The literature emphasizes the need for robust legal and policy frameworks to ensure data quality, protect patient privacy, mitigate workforce displacement, and foster public trust in AI systems [161,162]. These challenges highlight the complexity of responsibly embedding intelligent systems into clinical environments.

In particular, Char et al. [150] underline critical ethical risks associated with AI and ML, such as algorithmic bias, lack of fairness, and transparency, issues that must be addressed to ensure their equitable and safe use in healthcare. Legal accountability is also a major concern: as Naik et al. [163] note, assigning responsibility for clinical errors or adverse outcomes resulting from AI-driven decisions is both ethically and legally complex.

Therefore, while frameworks such as that proposed by Ali et al. represent significant progress in creating secure and scalable AI-enabled healthcare systems, their adoption must be accompanied by comprehensive ethical, legal, and regulatory strategies. In this regard, ensuring clear accountability through legislation like the European Union’s Artificial Intelligence Act, is an essential first step in establishing proper governance of ethical and legal responsibilities in AI-driven healthcare. The Act is the first attempt in creating a risk-based regulatory framework, which balance regulatory obligations according to the potential harm that AI systems pose to fundamental rights, security and welfare. High-risk applications demand strict oversight, including transparency requirements, human supervision and post-market monitoring. Traceability mechanisms become essential to retroactively determine accountability through the chain of stakeholders. Since accountability in AI-assisted decision-making is inherently distributed and context-dependent, it must be governed through a combination of legal, institutional, and procedural safeguards that are flexible enough to adapt to uncertainty while ensuring accountability and patient protection [88].

Moreover, in the healthcare context, responsibility is closely connected to other foundational principles, such as trust, justice, and autonomy. It is not a standalone concept but is often discussed in relation to systemic and ethical challenges. A responsibility gap emerges when it is unclear who should be held accountable for the actions or decisions of an AI system, especially in the event of harm [164,165]. This is frequently due to the evolving, opaque nature of AI technologies and the multiple stakeholders involved across development and clinical deployment.

This gives rise to responsibility diffusion, where responsibility becomes fragmented and uncertain among actors such as healthcare providers, developers, institutions, and patients [165,166,167]. To address these challenges, scholars have called for clear and adaptive frameworks that allocate responsibility effectively in cases of harm, particularly in high-stakes applications such as AI-based handwriting analysis [165,166].

Addressing these concerns is essential not only for ensuring patient safety and maintaining trust in healthcare systems, but also for enabling the sustainable and ethical adoption of intelligent technologies within clinical practice.

## 7. Ongoing Research

### 7.1. Integration with Wearable Devices and Smart Environments

Recent advancements in wearable medical technology have significantly transformed PD monitoring [20,168,169,170]. These devices overcome traditional clinical evaluation challenges, including subjectivity, sporadic assessments, and short-term observations, by offering objective, long-term monitoring, and comfort in patients’ homes. Wearable devices can capture objective data on tremor, bradykinesia, dyskinesia, gait, and other movements that might be missed in episodic evaluations. These systems typically incorporate inertial sensors (accelerometers and gyroscopes) and specialized algorithms to quantify motor features in real time. This approach overcomes the subjectivity of patient self-reports and the challenges of tracking symptoms like motor fluctuations via diaries or occasional clinic visits. As a result, interest in wearables for PD has grown rapidly, with clinicians and researchers exploring their potential to supply personalized treatments [168,169,170].

In January 2024, the National Institute for Health and Care Excellence (NICE) [171] updated its guidelines, endorsing five wearable systems for the remote monitoring of PD, listed in Table 2 [168,169,170].

However, integrating these devices into routine care presents some challenges. Persistent concerns about data accuracy can undermine clinician trust. Many tools lack independent validation, and evidence linking device data to better clinical outcomes remains limited. Usability and patient adherence are also critical; devices must be practical, comfortable, and easy-to-use. Multi-sensor systems can be burdensome over time, while single wearables (e.g., smartwatches) are generally more acceptable for daily use but, in some cases, less efficient for some movement symptoms. As a result, developers are working on enhancing user-friendliness (battery life, design, data clarity) while ensuring robust clinical validation. Widespread adoption also depends on meeting regulatory standards and demonstrating clear benefits in care decisions [168,169,170].

Beyond wearables, domestic smart environments offer a complementary approach for continuous, non-intrusive monitoring and care in PD. Smart environments refer to the integration of advanced technology in home environment in order to support and ameliorate human well-being, efficiency, and function. In-home sensors and “domotics” (smart home technologies) can passively track a patient’s activities and safety. For example, ambient sensors or camera systems can detect falls (a major risk in PD often under-reported by patients) and automatically notify caregivers or emergency services. A smart home can even recognize episodes like freezing of gait and respond with assistive cues—such as turning on floor lights to help the patient resume movement. Similarly, voice-activated systems could detect a patient’s sudden “off” episode and connect them to help (or adjust the environment) without the patient needing to reach for a device. This ambient monitoring strategy enhances patient safety and independence at home while relieving caregiver burden through automated supervision and timely alerts [172].

Both wearable devices and smart environments offer complementary data to handwriting monitoring, providing a multidimensional view of a patient’s behavior and health status. The integration of handwriting monitoring into these systems is a growing research focus. Embedding handwriting tasks into wearable devices allows researchers and clinicians to assess motor control in daily life situations.

### 7.2. Multimodal Data Fusion (Handwriting + Speech + Gait Analysis)

The complexity of neurological diseases like PD demands multimodal approaches that combine various forms of behavioral and biometric data [173,174]. Handwriting changes such as micrographia, stroke irregularities, and pressure variations have been well documented as early indicators of PD, as discussed previously. When these handwriting markers are combined with data from speech (e.g., vocal tremor, articulation deficits) and gait analysis (e.g., stride variability, postural instability), researchers achieve a richer, more holistic view of the disease [173,175].

In the speech domain, significant advancements have been made through the application of ML to voice analysis. Suppa et al. [175] demonstrated that automated voice analysis can objectively detect early voice impairment, often present in the prodromal phase of PD, with high diagnostic accuracy. Their study introduced a novel score (LR value) and confirmed its strong correlation with disease duration, motor severity, and cognitive decline. Notably, even early-stage drug-naive patients were distinguishable from healthy subjects, highlighting the value of speech biomarkers for early detection [175].

Similarly, Xu et al. [176] used facial landmark analysis during phonation tests to measure subtle motor impairments. Their findings revealed reduced movement across 49 facial points in PD patients, particularly in the eyes, eyebrows, and lips—corresponding with classical signs of “freezing mask” and reduced expressiveness. Such studies underscore the potential of integrating facial and speech analysis for early-stage diagnosis.

Furthermore, the integration of gait analysis into multimodal diagnostic systems is gaining importance. The study by Di Biase et al. [35] examined the use of technology for objective gait analysis in PD patients, revealing how gait disturbances evolve as the disease progresses. Early-stage gait alterations include reduced arm swing amplitude, decreased smoothness of locomotion, and increased interlimb asymmetry, which become more pronounced over time [177,178]. As the disease advances, more severe issues such as freezing of gait (FOG), motor fluctuations, reduced balance, and postural control emerge [177]. The study also highlighted that gait analysis using motion capture devices demonstrated high sensitivity and specificity (over 90%) in differentiating PD patients from healthy controls, underlining the potential of these technologies for objective assessment [35].

In conclusion, the fusion of handwriting, speech, and gait analysis holds great promise in enhancing the diagnostic sensitivity, tracking disease progression, and enabling earlier and more individualized interventions. While each modality shows significant potential, further research is required to integrate these data streams into unified diagnostic frameworks, offering more robust and clinically applicable tools for managing PD.

### 7.3. Real-World Deployment, Telemedicine Integration, and Non-Invasive Brain Stimulation

Telemedicine, defined as the remote delivery of healthcare services using telecommunications technology [179], has transformed access to care, particularly for chronic conditions like PD [172]. Beyond convenience, telemedicine offers a platform for continuous monitoring, rehabilitation, and personalized management. Telerehabilitation in PD has shown effectiveness not only in addressing motor impairments but also non-motor symptoms while improving patient compliance, underscoring its potential as a complementary approach to traditional care models [180].

Despite encouraging findings from pilot studies on multimodal monitoring in PD, real-world implementation remains challenging. Devices like STAT-ON™ exemplify early success in bridging this gap by enabling continuous, remote tracking of PD symptoms with validated clinical accuracy [20].

Telemedicine has emerged as a key platform for deploying such technologies, especially in underserved areas. The COVID-19 pandemic accelerated the adoption of remote healthcare, emphasizing the value of non-invasive, home-based monitoring solutions [180]. Recent study protocol, such as the TelePD study, explore integrating wearable sensors with virtual rehabilitation, demonstrating that balance and gait assessments can be effectively conducted via telehealth using instrumented mobility tests [181]. This approach supports personalized interventions and extends specialist care beyond traditional settings.

Integration into telemedicine workflows presents ongoing challenges. Ensuring data privacy under regulations is critical, as is achieving interoperability across devices and platforms. Additionally, algorithms must be robust against real-world variability, incomplete data, and environmental noise [170,178].

Handwriting analysis tools, especially those based on combined kinematic and non-linear dynamic features, show promise for remote diagnostics. As already discussed, studies like Ríos-Urrego et al. [58] demonstrated that simple tasks such as drawing spirals or writing standard sentences can yield high diagnostic accuracy even when performed on commercially available tablets, suggesting feasible scalability to home settings. Looking forward, hybrid telemedicine models, where patients routinely perform brief handwriting, voice, and mobility tasks at home, could enable AI-driven alerts for clinical review. This would enhance early intervention, continuous monitoring, and personalized treatment, improving outcomes while reducing healthcare burdens.

In parallel with AI-based handwriting biomarker research, emerging evidence supports the potential of non-invasive brain stimulation (NIBS) techniques, such as transcranial direct current stimulation (tDCS) and repetitive transcranial magnetic stimulation (rTMS), as complementary interventions in PD. These approaches have gained attention due to their ability to modulate cortical excitability and promote neuroplasticity, potentially enhancing motor learning and dual-task performance. Recent meta-analyses by Lee et al. suggest that anodal tDCS targeting the dorsolateral prefrontal cortex significantly improves dual-task motor and cognitive performance in PD, areas often compromised in this population [182]. Broeder et al. provide evidence that coupling writing training with atDCS over the primary motor cortex significantly enhances retention of handwriting skills, and continued motor learning in people with PD, regardless of medication state. Their trial demonstrated large effect sizes in improving writing amplitude both on digital tablets and traditional paper, suggesting that atDCS may help overcome some of the known limitations in skill generalization and retention in PD rehabilitation. Although dual-task performance during handwriting did not improve with stimulation, the intervention increased cortical inhibition, which may underlie the enhanced consolidation effects observed [183].

Such neuromodulatory tools could be further empowered by AI-based models to personalize stimulation parameters and monitor outcomes in real time. Additionally, Ng et al. underscore the importance of integrating AI into holistic, collaborative care ecosystems, highlighting patient engagement, clinician trust, and accessibility as essential pillars in overcoming sociotechnical barriers to implementation [184].

## 8. Conclusions

PD represents a chronic condition that requires an integrated approach throughout all stages of care. Beyond the well-known motor symptoms, such as bradykinesia, tremor, rigidity, akinesia, and freezing, PD also impacts fine motor skills, including handwriting abilities. Recent evidence highlights that handwriting impairments in PD extend far beyond micrographia. Instead, they encompass a broader dysfunction known as dysgraphia, reflecting complex disturbances in fluency, coordination, and motor planning. This conceptual shift carries important theoretical and practical implications: while letter size reduction is a visible marker, dynamic and kinematic variables, such as velocity, acceleration, and movement smoothness, offer more sensitive biomarkers for early diagnosis, disease monitoring, and therapeutic evaluation.

Traditional methods for handwriting analysis in PD, largely based on visual inspection and static measurements, have proven valuable but limited by subjectivity and inability to capture subtle motor deficits. The integration of AI has revolutionized this field, enabling automated detection and classification of PD-related handwriting impairments through the extraction of key spatial, temporal, and kinematic features. Supervised learning models, alongside advanced DL architectures such as CNNs, LSTMs, and hybrid approaches, have demonstrated high accuracy in distinguishing PD patients from healthy individuals. Despite the promise of AI to automate feature extraction, many high-performing systems still rely on manual design and domain expertise, particularly in the early stages of model development. This dependency poses a significant limitation for scalability and standardization across studies. Moreover, while CNNs have shown promise in processing handwriting images, their utility is often restricted to offline handwriting tasks, where dynamic temporal signals are not available. This limits their compatibility with online, real-time handwriting capture, a modality increasingly recognized for its diagnostic potential. Notably, combining various features, such as pressure and kinematic parameters, does not uniformly lead to improved performance; in some cases, the integration of multiple modalities introduces noise or redundancy. Recent studies suggest that pressure-derived dynamic features may offer superior discriminative power compared to image-based CNN outputs, even when the latter are extensively fine-tuned. These findings underscore the need for critical evaluation of the trade-offs between feature types and AI architectures and highlight that not all automated approaches yield equally effective outcomes in the detection of Parkinson’s disease.

AI-based handwriting analysis still faces challenges, including the need for large, diverse datasets and ensuring model interpretability in clinical settings. Its advantages, such as objectivity, scalability, and continuous monitoring potential, are undeniable. Future directions point towards integrating these AI tools with wearable devices, smart environments, and multimodal approaches within telemedicine frameworks. Such innovations promise to enhance personalized care, enabling real-time symptom tracking and proactive management of PD beyond traditional clinical boundaries.

## 9. Final Reflections and Future Directions

The integration of AI with handwriting analysis represents a critical inflection point in the clinical management of Parkinson’s Disease. This review not only highlights a novel diagnostic and monitoring approach but also proposes a paradigm shift in how subtle motor dysfunctions are conceptualized, as dynamic, measurable, and responsive to intervention. The findings underscore the potential for handwriting-based digital biomarkers to enhance early detection, personalize rehabilitation strategies, and enable continuous care through telemedicine platforms. Several methodological challenges remain, including the need for large-scale, demographically diverse datasets, robust model interpretability, and consensus on standardization of handwriting tasks and metrics. Future research should also explore multimodal data integration, combining handwriting with speech, gait, and neuroimaging features to construct comprehensive phenotypic profiles of PD. Furthermore, ethical considerations, particularly regarding algorithmic fairness and clinical accountability, must be addressed as these tools transition from research to practice. Ultimately, bridging the gap between computational innovation and real-world application will require interdisciplinary collaboration, regulatory clarity, and patient-centered design, elements essential to unlocking the full promise of AI-enhanced neurological care.

## Figures and Tables

**Table 1:** Conventional and advanced features characteristics in handwriting analysis.

Category	Feature Type	Examples
Conventional features [54]	Temporal	Writing duration, stroke duration
	Spatial	Stroke width, height, and length
	Kinematic	Velocity, acceleration, jerk
	Dynamic	Pressure, tilt, azimuth
Advanced features [54]	Entropy measures	Assessing randomness and irregularity of fine movements
	Signal-to-noise ratio (SNR)	Evaluating motor signal clarity via signal-to-noise ratio
	Empirical mode decomposition (EMD)	Decomposing signals into intrinsic mode functions
	Cepstrum analysis [103]	Identifying periodic patterns
	Sigma-lognormal models [104]	Neuromotor-based modeling of stroke velocity

**Table 2:** Characteristics of wearable devices for PD symptom monitoring approved by NICE in January 2024.

Device	Short Description
Parkinson’s KinetiGraph (PKG™)	Wrist-worn monitor for bradykinesia and dyskinesia episodes.
Kinesia 360™	Wrist-and-ankle sensor system linked to a smartphone app for tremor and movement tracking.
KinesiaU™	Smartwatch-based symptom tracker for patient self-monitoring via the Kinesia app.
PDMonitor^®^	Multi-sensor network (wearable on trunk and limbs) capturing gait, tremor, on/off fluctuations, etc.
STAT-ON™ [19]	Belt-worn inertial recorder logging on/off states, dyskinesia, falls, and gait parameters.

## Data Availability

Data are contained in the article and cited articles.
